# Cytomegalovirus-Specific Immunoglobulin G Is Associated With Chronic Lung Disease in Children and Adolescents from Sub-Saharan Africa Living With Perinatal Human Immunodeficiency Virus

**DOI:** 10.1093/cid/ciaa1757

**Published:** 2020-11-26

**Authors:** Dan Hameiri Bowen, Evgeniya Sovershaeva, Bethany Charlton, Cecilie Schive, Jon Odland, Grace McHugh, Tsitsi Bandason, Justin Mayini, Rashida A Ferrand, Louis-Marie Yindom, Sarah L Rowland-Jones

**Affiliations:** 1 Nuffield Department of Medicine, University of Oxford, Oxford, United Kingdom; 2 The Arctic University of Norway, Tromsø, Norway; 3 Department of Clinical Research, London School of Hygiene and Tropical Medicine, London, United Kingdom; 4 Biomedical Research and Training Institute, Harare, Zimbabwe

**Keywords:** cytomegalovirus, HIV, immunoglobulin G, lung disease

## Abstract

In a cross-sectional study of 296 children and adolescents from Zimbabwe living with perinatal human immunodeficiency virus, individuals with the top tertile of cytomegalovirus-specific immunoglobulin G titer had an increased odds of chronic lung disease (odds ratio, 3.33; 95% confidence interval, 1.37–8.85; *P* = .010).

Widespread use of combination antiretroviral therapy (cART) has increased the number of children who live with perinatal human immunodeficiency virus (PHIV) surviving into adolescence. However, there is growing evidence that despite cART, HIV in children is associated with multisystem chronic comorbidities and concomitant disability [1]. This is likely driven by chronic systemic immune activation, which cytomegalovirus (CMV) coinfection can exacerbate.

Studies from sub-Saharan Africa have shown that chronic lung disease (CLD) is a common comorbidity that affects about one-third of PHIV children aged ≥10 years [2]. Radiological findings are consistent with constrictive obliterative bronchiolitis (COB) as the main cause [3]. COB is typically characterized by ongoing airway inflammation, resulting in progressive tissue remodeling, fibrosis of the small airways, and lung function decline [4]. Pediatric COB in the Southern Hemisphere mostly occurs as a sequela of severe lower respiratory tract infections (LRTIs) [4]. As a common LRTI in infants living with HIV, the association of CMV with CLD is of particular interest. CMV is a common cause of pneumonitis in infants with PHIV and has been shown to exacerbate experimental pulmonary fibrosis in murine models [5]. In individuals living with HIV, CMV coinfection contributes to immune activation and inflammation-related morbidities, even in the context of virological suppression of HIV by cART [6].

In recent studies, our group described a high prevalence of CMV DNA in the plasma of children and adolescents from Zimbabwe with PHIV [7]. In this population, CMV viral load >1000 copies/mL was associated with reduced forced vital capacity, lower CD4 T-cell counts, and stunting. In the present cross-sectional study, we sought to determine the associations between both CMV-specific immunoglobulin G (IgG) titer and CMV plasma viremia with CLD as defined by airflow obstruction in a cross-sample of participants from the Bronchopulmonary Function in Response to Azithromycin Treatment for Chronic Lung Disease in HIV-infected Children (BREATHE) clinical trial [8].

## METHODS

We conducted a cross-sectional case-control study nested within the BREATHE trial [8] (NCT02426112). The trial recruited children and adolescents aged between 6 and 19 years living with PHIV from Malawi and Zimbabwe who had been taking cART for at least 6 months and with a diagnosis of CLD, defined as forced expiratory volume in 1 second (FEV_1_) *z* score less than –1 with lack of reversibility with salbutamol. The *z* scores were generated using Global Lung Function Initiative reference standards. Individuals with tuberculosis (TB), acute respiratory tract infections, or potentially fatal conditions at the time of screening were excluded. A comparison group matched for age (6–12 years and 13–19 years) and duration on cART (6 months to <2 years and >2 years) was recruited from HIV clinic attendees with FEV_1_ *z* scores >0 and no chronic cough in the past 3 months. First-thaw cryopreserved baseline plasma samples for participants recruited in Harare, Zimbabwe, were used for this study.

### Laboratory Methods

CMV-specific IgG levels were measured using the Abcam anti-CMV IgG human enzyme-linked immunosorbent assay kit per the manufacturer’s instructions. Samples were run in duplicate, and mean values per participant are reported in international units per milliliter. CMV-specific IgG levels were split into tertiles for the entire cohort and the CLD group.

Total viral nucleic acids were extracted from 200 µL plasma using the QIAamp MinElute virus spin kit (Qiagen, Hilden, Germany). Then, 100 µL of total viral nucleic acids were eluted and immediately stored at −80°C for subsequent testing. CMV detection was performed using the RealStar CMV quantitative polymerase chain reaction (PCR) kit v1.0 (Altona Diagnostics, Hamburg, Germany) per the manufacturer’s instructions. Samples were run on the QuantStudio 3 real-time PCR system in duplicate (Applied Biosystems, Foster City, CA). Samples were repeated when technical replicas were not concordant for CMV presence. CMV viral load is reported in international units per milliliter.

### Statistical Methods

Data were analyzed using R Studio (version 1.1.383). The mean and standard deviation were used to describe continuous variables, and categorical variables were described with proportions. Differences between study groups were assessed using Mann-Whitney *U* tests or χ ^2^ tests as appropriate. Weight-for-age and height-for-age *z* scores were calculated using British 1990 growth reference curves; all *z* scores that were less than –2 represented wasting and stunting, respectively. CLD was defined per the BREATHE protocol (FEV_1_ score less than –1) [8].

The association of CMV-specific IgG tertile and CLD was assessed using logistic regression. Linear regression was used to assess the association between both CMV measures and FEV_1_ *z* score. Participant age, sex, height-for-age *z* score, previous TB treatment, HIV viral load, and cART regimen were included as covariates in all models. A sensitivity analysis where enrollment into the CLD group was defined by FEV_1_ *z* score less than –1.64 was performed.

### Ethics

Consent from individuals within the BREATHE study was sought from the guardian and age-appropriate assent from the participant (for those aged <18 years). The Medical Research Council of Zimbabwe granted ethical approval for this substudy.

## RESULTS

A total of 241 cases and 55 controls were included in this study. A higher proportion of cases were female, reported previous treatment for TB, were stunted, and were wasted than in the control group (Supplementary Table 1). Cases had a lower mean CD4 T-cell count than the controls. There was no evidence of significant difference in HIV viral load or duration of cART between groups (Supplementary Table 1). Across both groups, the median (interquartile range) time on cART was years (4.15–8.42). The mean (standard deviation) CMV-specific IgG level was higher in the group with CLD than in the group without (48.4 ± 12.1 vs 39.7 ± 13.0, *P* < .001); 100% of participants were CMV seropositive. No control participants had detectable CMV DNA in plasma compared with 29 of 241 cases (12%).

Top and mid tertiles of CMV-specific IgG titer were significantly associated with increased odds of CLD compared with the bottom tertile (top-tertile odds ratio [OR], 3.33; 95% confidence interval [CI], 1.37–8.85; *P* = .010 and mid-tertile OR, 2.17; 95% CI, 1.60–4.55; *P* = .036; Table 1). CMV-specific IgG as a continuous measure was also associated with increased odds of CLD (OR, 1.05; 95% CI, 1.02–1.08; *P* = .003). CMV DNA in plasma was significantly associated with reduced FEV_1_ *z* score in cases (coefficient ± standard error = –0.30 ± 0.14; *P* = .028). Neither tertile nor CMV-specific IgG as a continuous measure were associated with FEV_1_ *z* score in the case group. In all analyses, duration of ART had no significant effect on model results. CMV-specific IgG titer negatively correlated with CD4 T-cell count in both groups. Spearman rank correlation coefficients are presented in Supplementary Figure 1. Only the association between CMV-specific IgG and FEV_1_ *z* score was modified in the sensitivity analysis (Supplementary Table 2).

**Table 1.** Factors Associated With Chronic Lung Disease (CLD) in the Case-Control Study and Factors Associated With Forced Expiratory Volume in 1 Second in Children With CLD (Linear Regression)

Variable	CLD Logistic Regression in Whole Cohort (n = 296)		Forced Expiratory Volume in 1 Second *z* Score Linear Regression in CLD Cases^b^ (n = 241)	
	Odds Ratio (CI, *P* Value) Univariable	Adjusted Odds Ratio (CI, *P* Value) ^a^	Coefficient ± SE, *P* Value Univariable	Coefficient ± SE, *P* Value Multivariable^a^
Middle tertile CMV-specific IgG	**2.34 (1.19–4.72, *P*** = **.015)**	**2.17 (1.06–4.55, *P*** = **.036)**	0.09 ± 0.12, *P* = .445	–0.05 ± 0.11, *P* = .655
Top tertile CMV-specific IgG	**5.19 (2.34–12.76, *P* <.001)**	**3.33 (1.37–8.85, *P* = .010)**	**–0.24** ± **0.11 *P* = .032**	–0.09 ± 0.12, *P*** = **.431
CMV-specific IgG (international unit)	**1.06 (1.03–1.09, *P*** < **.001)**	**1.05 (1.02–1.08, *P* = .003)**	**–0.01** ± **<0.01, *P* = .032**	< –0.01 ± < –0.01, *P*** = **.461
CMV DNA presence in plasma	N/A	N/A	**–0.28** ± **0.14, *P* = .047**	**–0.30** ± **0.14, *P* = .028**

Abbreviations: CI, 95% Confidence Interval; CLD, chronic lung disease; CMV, cytomegalovirus; IgG, immunoglobulin G; SE, standard error.

^a^All multivariable analyses include age, sex, height-for-age *z* score, previous tuberculosis treatment, combination antiretroviral therapy regime, and human immunodeficiency virus viral load as confounding variables. Tertile comparisons are compared to lowest tertile within the group compared. CMV DNA presence in plasma could not be included in logistic regression models because there were no cases in the control group. *P* < .05 are highlighted in bold.

^b^Linear regressions are performed in the CLD group only.

## DISCUSSION

As a common cause of LRTI in infants living with HIV, we hypothesized that CMV may be associated with CLD in the PHIV population [9]. Our results show that top tertile CMV-specific IgG titer is associated with obstructive lung disease, supporting previous associations between CMV presence in plasma and reduced lung function [7]. These findings contribute to our understanding of the association of CMV with HIV-1–associated airway disease in sub-Saharan Africa.

Infant coinfection with CMV and HIV leads to rapid disease progression and often pneumonitis that, along with systemic inflammation, may drive CLD [9]. CMV-specific IgG titer can be used as a putative marker of lifelong CMV exposure and increases with impaired control of the virus. CMV-specific IgG is associated with elevated immune activation markers and with the CD45RA+ CD27 T-cell memory phenotype, indicative of multiple rounds of restimulation [10, 11]. High antibody levels could represent increased exposure to CMV antigens before cART or persistent B-cell in activation in individuals with CLD. CMV viremia in the plasma of study participants is likely to indicate viral reactivation. The complete absence of CMV DNA in the control group is consistent with increased lifelong exposure to CMV within the case group.

This study is limited by its cross-sectional and associative design. Levels of CMV-specific IgG were generally high in the cohort, reflecting the overall burden of CMV infection in sub-Saharan Africa. The prevalence of CMV viremia and CMV viral load in the plasma of participants was lower than in recent reports, likely explained by more stable cART use in the BREATHE trial compared with previous studies [7]. As a result, the number of individuals with detectable CMV in plasma was small. Further work is required to determine whether CMV is a marker of impaired cellular immunity and/or the driver of the pathology described [12]. Lower respiratory tract samples would strengthen these findings.

In conclusion, we provide further evidence that CMV coinfection in individuals living with HIV is associated with CLD. We extend previous findings, reporting associations between CMV-specific IgG titer and CLD. HIV-associated comorbidities, such as CLD, represent a growing burden of disease in PHIV adolescents on stable cART. These results underline the need for future studies to assess causality and suggest that available CMV-specific antiviral drugs, such as valganciclovir, may be beneficial within this population of PHIV individuals at the time of acute infection or reactivation. Trials of such drugs would further help to determine the causality of CMV-associated pathogenesis.

## Supplementary Data

Supplementary materials are available at *Clinical Infectious Diseases* online. Consisting of data provided by the authors to benefit the reader, the posted materials are not copyedited and are the sole responsibility of the authors, so questions or comments should be addressed to the corresponding author.

ciaa1757_suppl_Supplementary_Materials

## Notes

***Acknowledgments.*** This work was supported through the Global Health and Vaccination Research (GLOBVAC] program of the Norwegian Research Council and HelseNord.

***Financial support.*** This work was supported by Norges forskningsråd (The Norwegian Research Council) and Helse Nord.

***Potential conflicts of interest.*** The authors: No reported conflicts of interest. All authors have submitted the ICMJE Form for Disclosure of Potential Conflicts of Interest. Conflicts that the editors consider relevant to the content of the manuscript have been disclosed.
