# Supplementary material for: Cytomegalovirus-Specific Immunoglobulin G Is Associated With Chronic Lung Disease in Children and Adolescents from Sub-Saharan Africa Living With Perinatal Human Immunodeficiency Virus
Source: Clin Infect Dis. 2020 Nov 26;73(1):e264–6. doi: 10.1093/cid/ciaa1757 (PMC8491836; doi:10.1093/cid/ciaa1757)
Supplement: ciaa1757_suppl_Supplementary_Materials [file ciaa1757_suppl_supplementary_materials.docx]

|  | Controls (n=55) | Cases (n=241) | P-Value |
| --- | --- | --- | --- |
| Age, years (Mean (SE)) | 15.2 (3.7) | 15.3 (3.3) | .962 |
| Female, N (%) | 18 (32.7%) | 128 (53.1%) | **.006** |
| Anthropomorphic Measures | | | |
| FEV_1_ Z-Score (Mean (SE)) | 0.6 (0.5) | -2.0 (0.7) | **< .001** |
| Height for Age z-Score (Mean (SE)) | -1.4 (0.8) | -2.0 (1.2) | **< .001** |
| Stunted (HFA z-score <-2) (Mean (SE)) | 13 (23.6%) | 110 (45.6%) | **.003** |
| Weight for Age z score ((Mean (SE)) | -0.9 (1.1) | -2.1 (1.5) | **.001** |
| Wasted (WFA z score <-2) | 8 (14.5%) | 123 (51%) | **.001** |
| CMV Measures | | | |
| CMV IgG (IU/mL) (Mean (SE)) | 39.7 (13.0) | 48.4 (12.1) | **.001** |
| CMV Viremia Present (N, %) | 0 (0.0) | 29 (12.0) | **.007** |
| CMV Plasma Viral load (IU/mL) (Median (IQR)) (n=29) | N/A | 419.5 (333.4-644.6) |  |
| HIV/TB Related Measures | | | |
| CD4 T Cell Count (cells/mm^3^) (Mean (SE)) | 669.8 (358.8) | 570.6 (349.1) | **.029** |
| CD4 < 350 (N, %) | 12 (21.8%) | 68 (28.2%) | .335 |
| CD4 > 350 (N, %) | 43 (78.2%) | 173 (71.8%) |  |
| Log_10_ HIV Viral Load/mL (Mean (SE)) | 2.5 (1.5) | 2.8 (1.5) | .14 |
| Uncontrolled HIV viral load, N (%)* | 17 (30.9%) | 100 (40.1%) | .147 |
| Duration of ART, years (Mean (SE)) | 6.6 (2.7) | 6.4 (3.0) | .411 |
| First Line ART (NRTI Based) (N, %) | 48 (88.9%) | 167 (69.6%) | **.004** |
| Second Line ART (PI Based) (N, %) | 6 (11.1%) | 73 (30.4%) |  |
| Previous treatment for TB (N, %) | 8 (14.5%) | 82 (34.0%) | **.005** |

Supplementary Table 1: Demographic, anthropomorphic and clinical characteristics by CLD status.

*Uncontrolled viral load defined as HIV viral load >1000 copies/ml. NRTI = Nucleoside Reverse-transcription inhibitor, PI= Protease Inhibitor, IU= International Units, N= Number, SE= Standard Error, IQR= Interquartile range, WFA= Weight for age, HFA= height for age)

B) Cases

A) Controls

Supplementary Figure 1: Spearman rank correlations between CMV, clinical and anthropomorphic variables measured in the study. A) PHIV control population B) PHIV CLD Cases

Where no significant correlation occurs (P> .05), squares are represented in white. Unsuppressed viral load defined as HIV viral load >1000 copies/ml. CD4 T Cell count = cells/mm^3^. Spearman rank correlations displayed separately for cases and controls. CMV Specific IgG tertile based on whole cohort.

|  | Odds Ratio (univariable) (OR, CI, P) | Odds Ratio Adjusted (OR, CI, P) |
| --- | --- | --- |
| Second CMV Specific IgG Tertile | 2.34 (1.19-4.72, P= .015) | 2.17 (1.06-4.55, P= .036) |
| Third CMV Specific IgG Tertile | 5.19 (2.34-12.76, P= <.001) | 3.33 (1.37-8.85, P= .010) |
| Sex (Female) | 0.43 (0.23-0.79, P= .007) | 0.56 (0.28-1.10, P= .095) |
| Height for age z-score | 0.54 (0.39-0.74, P= <.001) | 0.64 (0.45-0.89, P= .011) |
| Age in years | 1.01 (0.92-1.10, P= .834) | 0.98 (0.89-1.08, P= .706) |
| Having previously been treated for TB | 3.03 (1.44-7.20, P= .006) | 2.03 (0.89-5.11, P= .108) |
| Second line ART | 3.55 (1.56-9.57, P= .005) | 1.82 (0.72-5.29, P= .234) |
| Log_10_ HIV Viral Load (copies/ml) | 1.17 (0.96-1.44, P= .126) | 1.04 (0.83-1.30, P= .757) |

Supplementary Table 2: Factors associated with CLD (logistic regression)

Logistic regression results for all variables included in the regression model. Adjusted includes all variables included in table (n=291). OR= Odds ratio, CI= Confidence interval, P=P-value, TB = Tuberculosis, ART= Antiretroviral therapy.

| **Variable** | **Logistic Regression Results** | | **Linear Regression Results*** | |
| --- | --- | --- | --- | --- |
|  | Odds Ratio (CI, P) univariabe | Adjusted Odds Ratio (CI, P) ** | Coefficient ± SE, P univariable | Coefficient ± SE, P multivariable** |
| **Middle tertile CMV specific IgG** | **2.60 (1.27-5.47. P= .010)** | **2.32 (1.05-5.31, P= .041)** | 0.04 ± 0.12, P= .744 | -0.04 ± 0.12, P= .733 |
| **Top tertile CMV specific IgG** | **5.95 (2.59-15.09, P= <.001)** | **3.14 (1.21-8.80, P= .023)** | -0.14 ± 0.12 P=.234 | -0.11 ± 0.13, P= .413 |
| **CMV specific IgG (IU)** | **1.07 (1.04-1.10, P<.001)** | **1.05 (1.02-1.08, P= .004)** | -0.01 ± <0.01, P=.174 | <-0.01 ± <-0.01, P= .394 |
| **CMV DNA Presence in Plasma** | N/A | N/A | **-0.30** ± **0.14, P= .036** | **-0.31** ± **0.15, P= .033** |

Supplementary Table 3: Sensitivity analysis of main trial findings with CLD defined as FEV1 z-score <-1.64, n=159.

*Linear regressions are performed in the CLD group only. **All multivariable analysis includes age, sex, height for age z-score, previous TB treatment, cART regime and HIV viral load as confounding variables. Tertile comparisons are compared to lowest tertile within the group compared. CMV DNA presence in plasma could not be included in logistic regression models due to no cases in the control group. P= < .05 are highlighted in bold. CI= Confidence Interval, SE= Standard Error, IU= international units, p= p-value.
